# Apo‐state structure of the metabotropic glutamate receptor 5 transmembrane domain obtained using a photoswitchable ligand

**DOI:** 10.1002/pro.70104

**Published:** 2025-06-16

**Authors:** Yasushi Kondo, Caitlin Hatton, Robert Cheng, Matilde Trabuco, Hannah Glover, Quentin Bertrand, Fabienne Stierli, Hans‐Peter Seidel, Thomas Mason, Sivathmika Sarma, Friedjof Tellkamp, Michal Kepa, Florian Dworkowski, Pedram Mehrabi, Michael Hennig, Joerg Standfuss

**Affiliations:** ^1^ PSI Center for Life Sciences, Laboratory of Biomolecular Research Paul Scherrer Institute Villigen PSI Switzerland; ^2^ Institute for Nanostructure and Solid‐State Physics Hamburg Germany; ^3^ LeadXpro Biotech AG, Park Innovaare Villigen PSI Switzerland; ^4^ Max Planck Institute for the Structure and Dynamics of Matter Hamburg Germany; ^5^ PSI Center for Photon Science, Laboratory for Synchrotron Radiation and Femtochemistry Paul Scherrer Institute Villigen PSI Switzerland

**Keywords:** apo‐state, GPCR, light activation, X‐ray crystallography

## Abstract

Metabotropic glutamate receptor 5 (mGlu5) is implicated in various neurodegenerative disorders, making it an attractive drug target. Although several ligand‐bound crystal structures of mGlu5 exist, their apo‐state crystal structure remains unknown. Here, we study mGlu5 structural changes using the photochemical affinity switch, alloswitch‐1, in combination with time‐resolved freeze‐trapping methods. By X‐ray crystallography, we demonstrated that isomerizing alloswitch‐1 leads to its release from the binding pocket within a few seconds. The apo structure, determined at a resolution of 2.9 Å, is more comparable to the inactive state than to the active state. Our approach presents an accessible alternative to time‐resolved serial crystallography for capturing thermodynamically stable transient intermediates. The mGlu5 apo‐structure provides molecular insights into the ligand‐free allosteric pocket, which can guide the design of new allosteric modulators.

## 1 INTRODUCTION

G protein‐coupled receptors (GPCRs) constitute the largest superfamily of membrane proteins and are widely studied pharmacological targets (Sriram & Insel, 2018). Metabotropic glutamate receptors (mGlus), which belong to class C GPCRs, are mainly expressed in the brain and are involved in synaptic plasticity and neuronal excitability (Niswender & Conn, 2010). As a result, they play a critical role in many neurological disorders, including Parkinson's disease, Alzheimer's disease, and schizophrenia. mGlus form a constitutive dimer with an extensive N‐terminal extracellular domain (ECD) (Niswender & Conn, 2010). The endogenous ligand, L‐glutamate, binds to the ECD of the receptor and induces a conformational change that results in the asymmetric dimer formation of the C‐terminal transmembrane domain (TMD) (Koehl et al., 2019; Krishna Kumar et al., 2024) leading to the activation of downstream effector proteins through the cytoplasmic side of the TMD (Niswender & Conn, 2010).

The mGlu5 TMD adopts the canonical heptahelical configuration common to GPCRs. Within the TMD, a cavity formed by transmembrane helices (TMs) 2, 3, 5, 6, and 7 serves as a binding site for synthetic compounds that act as allosteric modulators (Doré et al., 2014). These modulators—classified as either negative allosteric modulators (NAMs) or positive allosteric modulators (PAMs)—alter the receptor's sensitivity to L‐glutamate (Niswender & Conn, 2010), and several small molecules targeting mGlus are currently under clinical investigation (Budgett et al., 2022).

**FIGURE 1:**
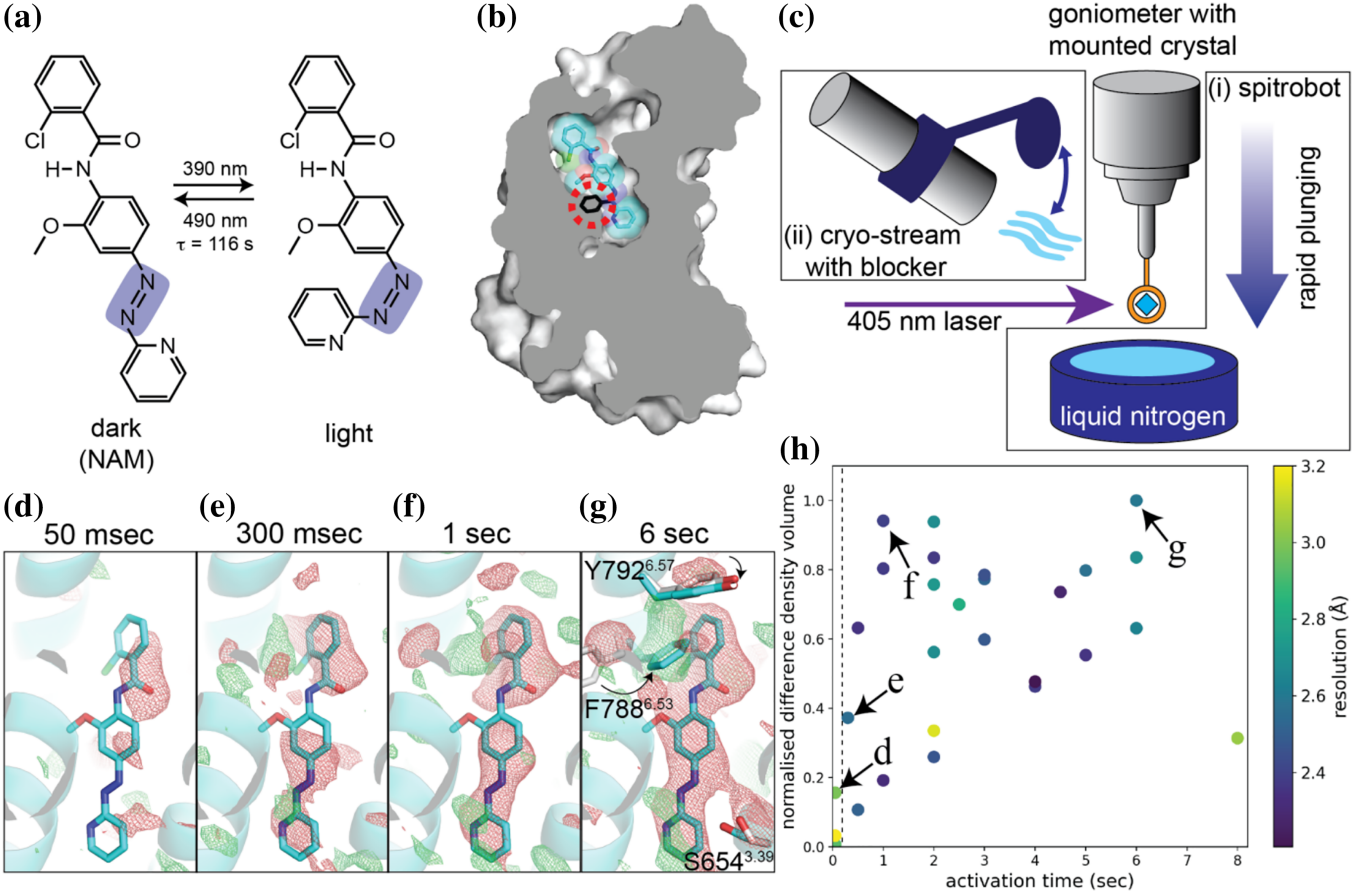
Photoisomerization of alloswitch‐1. (a) The chemical structures of alloswitch‐1. The azobenzene bond of alloswitch‐1 is highlighted in purple. (b) A structural superposition of the *cis*‐ and *trans*‐forms of alloswitch‐1 in the mGlu5 binding pocket. In the dark state, mGlu5 (gray) binds the *trans*‐form of alloswitch‐1 (cyan). The *cis*‐form of alloswitch‐1 (black) was generated by rotating the azo‐bond using Coot (Emsley et al., 2010). This speculative view illustrates the steric clash of the *cis*‐form with mGlu5, as experimental X‐ray data for the conformation are not available. (c) The set up for controlled illumination (i) using spitrobot and (ii) using an electronic cryo‐stream blocker. (d) Q‐weighted isomorphous difference peaks measured using the spitrobot, (e–g) and the annealing experiment. Q‐weighted difference electron density maps *F*_o_(light) − *F*_o_(dark) are contoured at 3.0 sigma and carved around alloswitch‐1. (h) The correlation between the activation time and the volume of the negative density around alloswitch‐1 in each X‐ray dataset. The datasets provided the q‐weighted difference maps shown in d–g are highlighted. For the comparison, the datasets obtained using the spitrobot are plotted at time zero and separated by the dotted line.

The field of photopharmacology has developed photoswitchable modulators for metabotropic glutamate receptors (Gómez‐Santacana et al., 2022). Among them, alloswitch‐1 acts as an mGlu5‐specific NAM that can be photoswitched through the introduction of an azobenzene moiety, which isomerizes upon light illumination (Figure 1a) (Pittolo et al., 2014). In its ground state, alloswitch‐1 adopts a *trans*‐form, which is shown to fit into the narrow binding pocket of the protein through a co‐crystal structure of mGlu5 and alloswitch‐1 (Figure 1b) (Nasrallah et al., 2021). Violet light (380–390 nm) converts the compound to the *cis*‐form, resulting in a ~20 times lower affinity for the protein upon light illumination (Pittolo et al., 2014). The isomerization of alloswitch‐1 azo‐bond, while anchoring the rest of the ligand in the pocket, causes a steric clash (Figure 1b). Computational docking and molecular dynamics simulation demonstrated a structural disruption of the binding pocket, which explains the observed reduction in affinity (Dalton et al., 2016). Light‐responsive behavioral changes upon the administration of the ligand have been demonstrated in various animal models ranging from zebrafish to mouse (Gómez‐Santacana et al., 2017; Notartomaso et al., 2024; Pittolo et al., 2014; Ricart‐Ortega et al., 2020). In principle, alloswitch‐1, therefore, acts as a photochemical affinity switch that can be used to modulate animal behavior.

Time‐resolved X‐ray crystallography studies protein dynamics using X‐ray Free Electron Lasers and next‐generation synchrotron sources, in combination with new approaches to trigger physiologically relevant reactions including rapid mixing approaches (Mehrabi et al., 2019), temperature jumps (Callender & Dyer, 2002) or chemically modifying substrates and ligands (Monteiro et al., 2021). We have recently shown how photochemical affinity switches can be used to determine the structures of short‐lived transition states within a time range of femto‐ to milliseconds (Standfuss, Weinert, et al., 2023; Wranik et al., 2023) and how such switches can be designed to target the orthosteric binding pocket of a class A GPCR in a time‐resolved serial crystallographic experiment (Glover, et al., 2024). However, this technology requires large amounts of crystals to be delivered to the X‐ray beam, which are not feasible for some protein targets, such as those that are difficult to express or crystallize less reproducibly, like many GPCRs. Other approaches to collect time‐resolved data with trigger systems, so that sample consumption is reduced, are needed to expand this method to a wider range of protein samples.

Rapid cryo‐cooling of crystals is an alternative method to room temperature serial crystallography (Mehrabi et al., 2023). The time resolution is currently limited to >50 ms due to the minimal delay time of the freezing device, and cooling can make crystals non‐isomorphous; however, a single crystal can be enough to determine a structure, making this method the preferred option for proteins that can only be produced in small quantities. Here, we used freeze‐trapping methods in conjunction with a photochemical affinity switch to investigate the structural changes in the TMD of the mGlu5 receptor. Negative difference density in the isomorphous difference map, obtained by comparing X‐ray diffraction data before and after light exposure, suggests successful isomerization of the ligand within the mGlu5 binding pocket. This observation is consistent with previous experiments demonstrating an enhanced dissociation rate of alloswitch‐1 upon light illumination (Ricart‐Ortega et al., 2020). The obtained apo‐state structure shows how an allosteric GPCR binding pocket conformationally adapts to the release of a negative modulator, including a shift of TM5 implicated in receptor activation.

## 2 RESULTS

To capture the sub‐second intermediates of mGlu5 after the photoisomerization of alloswitch‐1 in the allosteric binding pocket, we added a laser diode to the spitrobot (Mehrabi et al., 2023) to couple light activation and rapid crystal freezing. The spitrobot flash‐freezes a crystal with a minimal delay of approximately 50 ms after the onset of light exposure, while it takes another 50–500 ms for the crystal to reach the liquid nitrogen temperature, depending on the size of the crystal (Mehrabi et al., 2023).

We collected a 2.5 Å X‐ray diffraction dataset with a crystal frozen by the spitrobot without laser exposure as the reference dark state. We screened crystals treated under various activation conditions and obtained a 2.9 Å resolution dataset from a single crystal activated for 5 ms. The q‐weighted *F*_o_(light) − *F*_o_(dark) difference map clearly shows negative density at alloswitch‐1, indicative of alloswitch‐1 displacement (Figure 1d). However, the relatively low resolution and low activation level made the interpretation of the structural changes difficult. Doubling the light exposure time to 10 ms, to achieve a higher activation level, resulted in crystals with lower resolution quality (>3.5 Å) and shortening of the unit cell b‐axis (~39 Å instead of ~43 Å). Furthermore, light exposure longer than 10 ms yielded no diffraction patterns.

We next performed freeze‐trapping experiments at the X10SA (PXII) beamline at the Swiss Light Source. Here, frozen crystals were mounted on a goniometer, where a laser was pre‐aligned at the center of the cryoloop (Figure 1c). By blocking the cryostream with a cryoblocker (Giraud et al., 2008), a cryo‐cooled crystal was annealed at room temperature, while the pre‐aligned laser illuminated it to initiate photoswitch isomerization. After a defined illumination time, the crystal was re‐cooled by removing the cryoblocker to trap the photoactivated structure and reduce the radiation damage during X‐ray diffraction experiments. Since cooling by the cryostream occurs on the order of hundreds of milliseconds (Teng & Moffat, 1998), we focused on obtaining a steadily activated state using a weaker laser compared to the spitrobot experiment. We screened crystals at various illumination times, ranging from 300 ms to 8 s, before collecting X‐ray data. Measurement of a crystal without laser exposure provided a reference dataset at 2.3 Å resolution (Table 1). The dark structure was virtually identical to the previously reported alloswitch‐1‐bound mGlu5 crystal structure (PDB ID: 7P2L; Cα‐RMSD 0.17 Å) (Nasrallah et al., 2021).

**TABLE 1:** Data collection and refinement statistics.

	Dark structure	Light structure
Data collection		
Space group	C 1 2 1	C 1 2 1
Unit‐cell parameters		
*a, b, c* (Å)	143.221, 43.363, 82.642	143.160, 42.851, 82.765
*α*, *β*, *γ* (°)	90.00, 99.86, 90.00	90.00, 99.72, 90.00
Resolution^a^ (Å)	49.31–2.33 (2.58–2.33)	49.40–2.60 (2.81–2.60)
*R*_merge_	0.082 (0.907)	0.092 (0.973)
Completeness (%)		
Spherical	67.1 (13.1)	68.9 (16.3)
Ellipsoidal	86.8 (56.9)	87.6 (53.9)
Multiplicity	3.3 (3.3)	3.4 (3.4)
I/σI	8.8 (1.4)	8.5 (1.2)
CC_1/2_	0.997 (0.618)	0.998 (0.474)
Best/worst diffraction limits after cut‐off	2.331/3.071	2.595/3.894
Extraporation		
Resolution (Å)		19.93–2.90 (2.95–2.90)
*R*_iso_		0.20 (0.355)
CC_iso_		0.94 (0.663)
Refinement		
Resolution (Å)	49.31–2.33 (2.41–2.33)	19.93–2.90 (3.00–2.90)
No. reflections	14,590 (128)	9587 (603)
*R*_work_/*R*_free_	0.229/0.271	0.284/0.302
No. atoms	3401	3349
Amino acid residues	414	415
alloswitch1/lipid/Ion	1/5/6	0/4/4
Solvent	22	17
Rotamer outliers (%)	1.16	6.41
RMSD bond lengths (Å)	0.0034	0.0020
RMSD bond angles (°)	0.61	0.48
Average B factor (Å^2^)	50.9	49.3
Clash score	3.33	5.12
Ramachandran favored/outliers (%)	97.01/0.00	95.04/0.50
PDB ID	9HC0	9HC3

^a^ Values of the highest resolution shell are given in parentheses.

**FIGURE 2:**
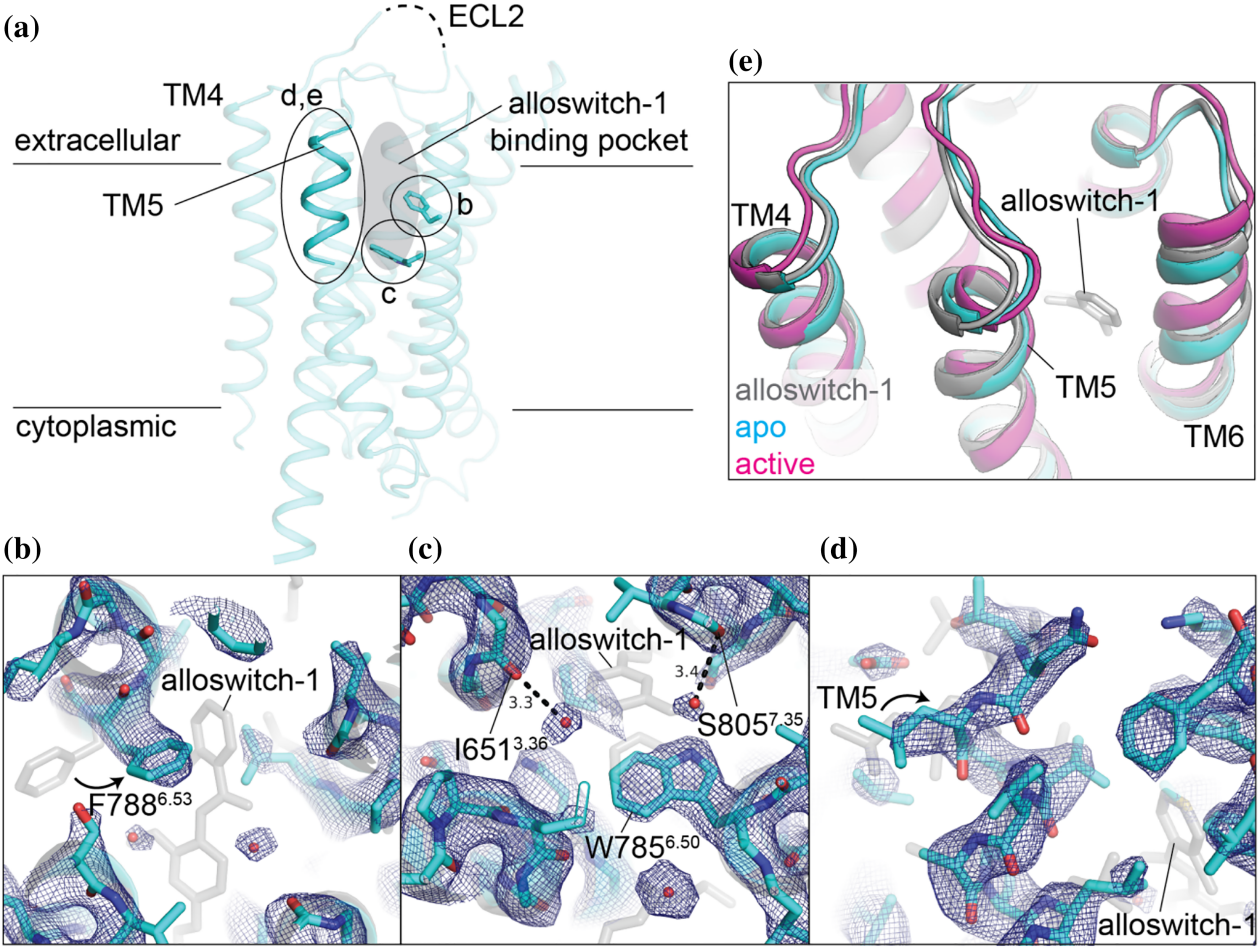
The apo‐state structure of mGlu5 TMD. (a) Overall structure of apo‐state mGlu5. Regions showing conformational changes (b) Phe788^6.53^, (c) Trp785^6.50^, and (d) TM5 are highlighted on the model. (b–d) Extrapolated map of local structural movements showing the apo‐state (cyan) and the dark state (gray) models. The extrapolated map is contoured at 1.5 sigma and carved 2.0 Å around structural models. (e) Highlighting the TM5 conformations among alloswith‐1 bound, apo, and active states (PDB ID: 6N51 chain B) (Koehl et al., 2019) by aligning the structures using TM1, 2, 3, 4, and 7.

We used the reference dark dataset for the calculation of q‐weighted *F*_o_(light) − *F*_o_(dark) difference maps and the extrapolation of structure factors for the active state using Xtrapol8 (De Zitter et al., 2022). Analysis of these data showed a clear time‐dependent increase in the negative density around alloswitch‐1 (Figure 1e–h) suggesting its release from the binding pocket. Longer exposure to the laser (8 s) produced a crystal with a shorter b‐axis and poor diffraction. We chose an X‐ray dataset collected after 6 s of activation for further analysis because this dataset had a high activation level of 60%, while maintaining a resolution of 2.6 Å (Figure 1g,h). Extrapolated density maps showed no density of alloswitch‐1 (Figure 2). The structural model refined against these data is hereafter referred to as the apo‐state.

The overall structure of the mGlu5 apo‐state was similar to the prototypical NAM‐bound structure (PDB ID: 4OO9; Cα‐RMSD of the 7‐TM helices: 0.37 Å) (Doré et al., 2014). Furthermore, our apo‐state crystal structure was more comparable to the ligand‐free chain in the full‐length mGlu5 cryo‐EM structure (PDB ID: 8TAO chain A; Cα‐RMSD of the 7‐TM helices: 1.06 Å) than to the PAM‐bound molecule (chain B; Cα‐RMSD: 1.27 Å) (Krishna Kumar et al., 2024). However, structural rearrangement of the protein to adapt to ligand displacement was evident in the apo‐state structure (Figure 2). The Phe788^6.53^ sidechain rotates toward the ligand pocket to a position with which the chlorophenyl group of *trans*‐alloswitch‐1 would clash in the dark state (Figure 2b). This sidechain conformation is consistent with computational docking models with various allosteric modulators (Dalton et al., 2016; Hellyer et al., 2020) as well as with other NAM‐bound crystal structures (Christopher et al., 2019; Doré et al., 2014). Negative density consistently appeared on Ser654^3.39^ and Tyr792^6.57^ in a series of isomorphous difference maps (Figure 1e–g). In contrast, the sidechain of the highly conserved conformational switch, Trp785^6.50^, maintains its orientation (Figure 2c). The extra densities observed adjacent to the mainchain carbonyl groups of Ile651^3.36^ and Ser805^7.35^ were interpreted as water molecules (Figure 2c). The water near Ile651^3.36^ is positioned similarly to that observed in an M‐MPEP‐bound structure (Christopher et al., 2019), while the water adjacent to Ser805^7.35^ occupies the space where the oxygen atom of the alloswitch‐1 anisol group is located. Collectively, these rearrangements contribute to a tilt of the extracellular end of TM5 toward the empty allosteric binding pocket (Figure 2d,e).

## 3 DISCUSSION

We applied freeze‐trapping methods combined with a photochemical affinity switch to study conformational changes upon ligand release from the mGlu5 receptor (Movie S1). We tested two methods to rapidly cryo‐cool crystals after light activation: a modified spitrobot (Mehrabi et al., 2023) for quick plunging into liquid nitrogen and controlled blockage of the cryo‐steam directly at a synchrotron beamline. Both methods successfully produced isomorphous difference maps indicating ligand displacement in mGlu5 with time resolution down to the millisecond range. However, despite computational docking and MD simulation indicating a possible binding mode for *cis*‐alloswitch‐1 in the mGlu5 pocket (Dalton et al., 2016), we did not observe corresponding electron density for the isomerized ligand in the photoactivated datasets.

GPCRs are particularly difficult to crystallize without ligands due to their flexible nature (García‐Nafría & Tate, 2021), which plays a key functional role. Diffusion‐based methods for rapid ligand mixing are thus unsuitable for time‐resolved experiments on GPCRs, and, with the exception of rhodopsins (Gruhl et al., 2023), GPCRs are not directly activatable by light. Our study suggests that freeze‐trapping experiments combined with photoswitchable ligands are an accessible and cost‐effective alternative to time‐resolved serial crystallography for capturing thermodynamically stable transient intermediates in the millisecond–second range.

We resolved the apo‐state structure of a GPCR at crystallographic resolution, and the model is very similar to that of NAM‐bound mGlu5 (Doré et al., 2014). It is important to note that our crystallization construct contains only the isolated TMD. Nevertheless, this finding is consistent with recent cryo‐EM studies of full‐length mGlu5 (Krishna Kumar et al., 2024), where the ligand‐free TMD within the homodimer exhibits a conformation similar to our apo‐state crystal structure, whereas the PAM‐bound TMD undergoes a conformational shift toward the active state. We acknowledge that in the full‐length receptor, interactions involving the CRD, ECLs, and inter‐TM contact within the dimer may further modulate the receptor conformation.

In addition to the rotation of the Phe788 sidechain to the ligand binding pocket, we observed a clear shift in the extracellular end of TM5 after ligand release (Movie S1). Given that changes in TM5 and the connected extracellular loop (ECL2) are critical for communication with the orthosteric site and the formation of an active mGlu5 dimer (Koehl et al., 2019), it seems likely that an induced fit upon the binding of NAM to the allosteric pocket prevents the receptor from adopting the active conformation necessary for signal transduction. In the absence of NAM, TM6, following TM5, is ready to form the asymmetric dimer interface, a critical step toward G protein activation (Krishna Kumar et al., 2024).

Four allosteric drugs targeting GPCRs (Cinacalcet, Ticagrelor, Ivermectin, and ATx‐201) have been approved, with many more in clinical trials (Shen et al., 2023). Allosteric modulators offer significant advantages as therapeutic agents (Thal et al., 2018). Their binding sites tend to be more variable across receptor subtypes compared to orthosteric sites, enabling greater selectivity and potentially reducing off‐target effects. In addition, allosteric modulators can fine‐tune receptor activity rather than fully activating or inhibiting the receptor, as is common with orthosteric ligands. The combination of allosteric modulation with light‐switchable compounds may allow for even more precise control of receptor function. Our structure of the ligand‐free allosteric pocket provides a framework for optimizing the affinity and functional properties of this promising class of compounds.

## 4 MATERIALS AND METHODS

### 4.1 Sample preparation

A plasmid encoding the mGlu5‐StaR (569–836)‐T4L construct (Doré et al., 2014) was cloned by Genscript. mGlu5 was overexpressed in Hi5 cells and purified and crystallized with alloswitch‐1 (Abcam ab147022) as previously described (Nasrallah et al., 2021), using a crystallization buffer containing 20–30% PEG300, 0.1–0.2 M diammonium hydrogen phosphate, and 1% 1,6‐hexanediol.

### 4.2 Crystal activation

A continuous 405 nm 300 mW laser (Thorlabs LP405‐MF300) was attached to the spitrobot (Mehrabi et al., 2023) with a benchtop current controller (Thorlabs LDC205C). The 405 nm wavelength laser with a spot size of 200 × 200 μm^2^ was pre‐aligned to a cryoloop mounted to the spitrobot. After harvesting, crystals were mounted on the spitrobot and flash‐frozen before illumination at room temperature. This freeze‐and‐thaw step was introduced to make results consistent with cryostream experiments. The annealing procedure removed the excess amount of LCP from the cryoloop and produced well‐diffracting crystals. After annealing, an mGlu5 crystal was illuminated by the 200 mW laser (measured at the sample) for 5, 10, 20, 30, or 40 ms, controlled by a RIGOL Waveform Generator DG4102, before being flash‐frozen in liquid nitrogen. For annealed crystals, we flash‐froze the crystals using the spitrobot and brought them back to room temperature before activation.

For the activation while annealing experiment using cryostream, the setup at PXII beamline at Swiss Light Source was used. mGlu5 crystals were harvested and flash‐frozen by liquid nitrogen. Frozen crystals were mounted on the goniometer by the TELL sample changer and annealed by an electronic cryoblocker for a set time. During this annealing period, activation was performed by light exposure with a 2 mW, 405 nm wavelength cw laser diode with a spot size of 100 × 80 μm^2^ at the sample position. X‐ray diffraction data were immediately collected after activation.

### 4.3 Data collection and processing

X‐ray diffraction data were collected at 100 K using a wavelength of 1 Å at the p14 beamline, EMBL@PetraIII (spitrobot) and the X10SA (PXII) beamline, Swiss Light Source (cryostream). Datasets were indexed, integrated, and scaled by XDS (Kabsch, 2010). The datasets were anisotropically scaled by the STARANISO server at https://staraniso.globalphasing.org/. Q‐weighted difference maps and extrapolated structure factors were calculated using Xtrapol8 with the q‐weighting and *F*_extra_ method without automatic refinement of the model, and the resolution cutoff was set to fulfill *R*_iso_ <0.40 and CC_iso_ >0.60 during extrapolation (De Zitter et al., 2022). The activation level was estimated by the *difference‐map* method. Structural models were edited using COOT (Emsley et al., 2010) and the models were refined using phenix.refine (Liebschner et al., 2019). Figures with structural models and electron density maps were prepared using PyMOL (Schrodinger LLC).

### 4.4 Map analysis

To quantify the difference in electron density maps, first an ROI was defined. The ROI was defined as a 3 Å radius around the ligand. The ROI was then sampled at 10000 random positions inside the ROI using the gemmi function to interpolate values. Values obtained this way were thresholded to 3 sigma, replacing under‐threshold values with 0. Then, the mean of the values was calculated.

## AUTHOR CONTRIBUTIONS

**Yasushi Kondo:** Investigation; validation; visualization; writing – original draft. **Caitlin Hatton:** Investigation; methodology; writing – review and editing. **Robert Cheng:** Investigation; writing – review and editing. **Matilde Trabuco:** Investigation; writing – review and editing. **Hannah Glover:** Investigation; writing – review and editing. **Quentin Bertrand:** Investigation; writing – review and editing. **Fabienne Stierli:** Investigation; writing – review and editing. **Hans‐Peter Seidel:** Investigation; writing – review and editing. **Thomas Mason:** Writing – review and editing. **Sivathmika Sarma:** Writing – review and editing. **Friedjof Tellkamp:** Methodology; writing – review and editing. **Michal Kepa:** Methodology; writing – review and editing. **Florian Dworkowski:** Methodology; writing – review and editing. **Pedram Mehrabi:** Investigation; methodology; writing – review and editing; supervision; conceptualization; funding acquisition. **Michael Hennig:** Writing – review and editing; supervision; conceptualization; funding acquisition. **Joerg Standfuss:** Writing – review and editing; supervision; conceptualization; funding acquisition.

## CONFLICT OF INTEREST STATEMENT

Some authors are employees of LeadXpro Biotech AG, a company offering services for GPCR drug design and develops own lead compounds. The other authors declare no financial interests.

## Supporting information


**Movie S1.** Conformational changes of the mGlu5 TMD. Morphing from the alloswitch‐1 bound dark state to the apo‐state obtained by light activation of the ligand, followed by morphing from the apo‐state to the active state (PDB ID: 6N51 chain B) (9) is shown. The color coding corresponds to the one described in Figure 2e.

## Data Availability

Coordinates and structure factors have been deposited in the Protein Data Bank database under accession codes 9HC0 (dark) and 9HC3 (light).
